# Ultrasound-mediated augmented exosome release from astrocytes alleviates amyloid-β-induced neurotoxicity

**DOI:** 10.7150/thno.52436

**Published:** 2021-02-25

**Authors:** Zhiting Deng, Jieqiong Wang, Yang Xiao, Fei Li, Lili Niu, Xin Liu, Long Meng, Hairong Zheng

**Affiliations:** Paul C.Lauterbur research center for biomedical imaging, Shenzhen Institutes of Advanced Technology, Chinese Academy of Sciences, Shenzhen, People's Republic of China. 1068 Xueyuan Avenue, Shenzhen University Town, Shenzhen, 518055, China.

**Keywords:** ultrasound stimulation, astrocytes, exosomes, Alzheimer's disease, iTRAQ

## Abstract

**Background:** Extracellular vesicles, including exosomes, are secreted by a variety of cell types in the central nervous system. Exosomes play a role in removing intracellular materials from the endosomal system. Alzheimer's disease (AD) is caused by an overproduction or reduced amyloid-beta (Aβ) peptide clearance. Increased Aβ levels in the brain may impair the exosome-mediated Aβ clearance pathway. Therapeutic ultrasound stimulation demonstrated its potential for promoting Aβ degradation efficiency in clinical trials. However, the underlying mechanism of ultrasound stimulation is still unclear.

**Methods:** In this study, astrocytes, the most abundant glial cells in the brain, were used for exosome production. Post insonation, exosomes from ultrasound-stimulated HA cells (US-HA-Exo) were collected, nanoparticle tracking analysis and protein analysis were used to measure and characterize exosomes. Neuroprotective effect of US-HA-Exo in oligomeric Aβ_42_ toxicated SH-SY5Y cells was tested. Cellular uptake and distribution of exosomes were observed by flow cytometry and confocal laser scanning microscopy. Focused ultrasound (FUS) with microbubbles was employed for blood-brain-barrier opening to achieve brain-targeted exosome delivery. After US-HA-Exo/FUS treatment, amyloid-β plaque in APP/PS1 mice were evaluated by Aβ immunostaining and thioflavin-S staining.

**Results:** We showed that ultrasound resulted in an almost 5-fold increase in the exosome release from human astrocytes. Exosomes were rapidly internalized in SH-SY5Y cells, and colocalized with FITC-Aβ_42_, causing a decreased uptake of FITC-Aβ_42_. CCk-8 test results showed that US-HA-Exo could mitigate Aβ toxicity to neurons *in vitro*. The therapeutic potential of US-HA-Exo/FUS delivery was demonstrated by a decrease in thioflavin-S-positive amyloid plaques and Aβ immuno-staining, a therapeutic target for AD in APP/PS1 transgenic mice. The iTRAQ-based proteomic quantification was performed to gain mechanistic insight into the ultrasound effect on astrocyte-derived exosomes and their ability to alleviate Aβ neurotoxicity.

**Conclusion:** Our results imply that US-HA-Exo have the potential to provide neuroprotective effects to reverse oligomeric amyloid-β-induced cytotoxicity *in vitro* and, when combined with FUS-induced BBB opening, enable the clearance of amyloid-β plaques *in vivo*.

## Introduction

The pathophysiological process of Alzheimer's disease (AD) is thought to begin many years before the diagnosis of AD 1. Before the formation of plaques, amyloid-beta (Aβ)-induced neuronal dysfunction exists 2. Developing strategies to reduce excess deposition of the neurotoxic protein and halt the related pathological changes is of paramount importance for Aβ clearance 3.

Recent studies have shown that failure in exosome production occurs in both AD mouse and human brains, and exosome pathway dysfunction plays a primary role in brain pathologies 4-7. Disruption of interdependent endosomal-exosomal-lysosomal systems may contribute to amyloidogenic Aβ precursor protein processing, leading to a higher risk of AD disease development 8, 9. Endosomal material released into the extracellular space via exosomes is an important mechanism by which neurons remove debris. Therefore, failure of efficient exosome production and release can impair this process, resulting in endosomal pathway disturbances 10. The imbalance in the production and clearance of Aβ is the main cause of AD. Therefore, increasing exosome production may be a therapeutic approach for AD.

Exosomes are nano-sized vesicles secreted by cells, packed with information, including signaling proteins as well as coding and regulatory RNAs, and can be taken up by target cells, thereby facilitating the transfer of multilevel information 11. Besides being beneficial for cell-to-cell delivery of RNA, proteins, and lipids 12, exosomes carry signaling information required to regulate neural circuit development 13. Recent studies also suggested that exosomes contribute to nerve regeneration, synaptic function, and also the clearance of Aβ from the brain 12, 14. Environmental changes such as laser, heat, and hypoxia could alter exosome production 15, 16. Recently, exogenous electric stimuli, magnetic fields, or ultrasound have been used as new AD therapy approaches 17, 18. Among the various commonly used external stimuli, ultrasound is advantageous due to its noninvasive nature and deep tissue penetrability. Ultrasound stimulation has shown great potential in AD therapy in animal models 19, 20. However, its effect on exosome release and the precise Aβ clearing mechanisms remain elusive.

Brain-targeted delivery of exosomes for neuronal diseases is hampered by the blood-brain barrier (BBB), limiting their efficient concentration in the brain 21. Previously, focused ultrasound (FUS) has shown great potential in safely opening the BBB in AD patients in a clinical trial 22. Delivery of therapeutics with FUS and microbubbles constitutes a safe and noninvasive strategy for brain targeted therapy 23.

In this study, ultrasound were used for the mechanical stimulation of astrocytes to examine its effect on exosome production. *In vitro* and *in vivo* models of AD were implemented to investigate the neuroprotective potential of astrocyte-derived exosomes in neurons. Combined with FUS-induced BBB opening, a noninvasive, brain-targeted exosome delivery in APP/PS1 mice was achieved. We demonstrated, for the first time, the increased release of exosomes from astrocytes upon mechanical ultrasound stimulation, and more significantly, amyloid clearance by the astrocyte-derived exosomes in APP/PS1 transgenic mice. We believe that mechanistic insights into possible ultrasound stimulation mechanisms gleaned from our investigation could be exploited in future therapeutic approaches for AD.

## Materials and Methods

### Cell culture

Human astrocytes (HA) and human neuroblastoma (SH-SY5Y) cells were purchased from the American Type Culture Collection, and cultured in Dulbecco's Modified Eagle's Medium (DMEM) (Corning, USA) supplemented with 10% fetal bovine serum (FBS, Gibco) and 1% penicillin/streptomycin (Sigma, USA). Cultures were maintained at 37 °C in a humidified atmosphere of 5% CO_2_.

### Reagents

The sources of various reagents were as follows: Thioflavin-S (Th-S) (Sigma, USA), PKH26 Red Fluorescent Cell Linker Kit (Sigma Aldrich, USA), DiR (Beyotime, China), Amyloid-β_42_ peptides (GL Biochem, China, 95% purity), and FITC-labeled amyloid-β_42_ peptides (GL Biochem, China, 95% purity), Protease Inhibitor Cocktail Set III, EDTA-Free (Calbiochem, USA).

### Animals

Male APP/PS1 mice and age-matched wild-type littermates were obtained from Nanjing University (Nanjing, China). Male BALB/c mice were obatined from Beijing Vital River Laboratory Animal Technology (Beijing, China). All animal procedures were approved by the Animal Care Committee of Shenzhen Institutes of Advanced Technology, Chinese Academy of Sciences. Animals were kept on a 12 h light/dark cycle and given ad lib access to food and water as per standard operating procedures.

### *In vitro* ultrasound stimulation for exosome production

Ultrasound stimulation was generated by a transducer at 1-MHz working frequency with a 20% duty cycle. The spatial-peak temporal-average intensity (ISPTA) was 280 mW/cm^2^. Degassed water was used to cover the area between the transducer and the cell culture plate to maximize ultrasound transmission. Astrocytes were subjected to multiple ultrasound stimulations with 3 min duration per spot. The total sonication time of ultrasound stimulation was 15 min. Seventy-two hours later, the cell culture supernatant was harvested and centrifuged to collect exosomes.

### Exosome isolation

Exosomes were harvested from cell culture supernatants. Three days before ultrasound stimulation, the medium was replaced with DMEM plus 10% exosome-depleted FBS (Vivacell, Germany). After ultrasound stimulation, the cells were incubated for three more days and allowed to reach about 80% cell confluency. The culture media were collected and centrifuged sequentially at 300×g for 10 min, 2,000×g for 30 min, and 10,000×g for 30 min at 4 °C to remove cellular debris. Exoquick-TC (System bioscience, USA) was added to the supernatant at 1:5 ratio (V/V) and incubated overnight at 4 °C to precipitate exosomes followed by centrifugation at 3000×g for 60 min. The pellet was resuspended in PBS and centrifuged at 12000×g for 60 min to collect exosomes.

### Characterization of exosomes

#### Transmission electron microscopy (TEM)

For negative-staining, purified exosomes were fixed in 2% (v/v) paraformaldehyde for 5 min at room temperature. Subsequently, suspensions were applied to formvar/carbon-coated grids (200 mesh) for 3 min and negatively stained with 2% uranium acetate. Excess uranyl acetate was removed with filter paper. The grids were examined under a transmission electron microscope at 120kV (JEM-1200EX, JEOL Ltd., Japan).

#### Nanoparticle tracking analysis (NTA)

The number and size of the exosomes were directly tracked using the NS300 instrument (Malvern Instruments Ltd., Worcestershire, UK) equipped with a 405 nm laser and a high-sensitivity CMOS camera. In this analysis, particles were automatically tracked and sized based on Brownian motion and the diffusion coefficient. The exosome pellets were resuspended and diluted in PBS to obtain a concentration within the recommended range (1 × 10^7^-1 × 10^9^ particles/mL) and vortexed for 1 min. The samples were loaded into the sample chamber at ambient temperature. One 60-s video was acquired for each sample. The videos were subsequently analyzed with the NTA3.2 software, which identified and tracked each particle's center under Brownian motion to measure the average distance the particles moved on a frame-by-frame basis.

#### Western blotting

Exosome samples were homogenized in RIPA lysis buffer with protease inhibitor cocktail and PMSF, and vortexed every 5 min for 30 min to lyse the exosomes on ice. Subsequently, lysates were centrifuged at 12,000g for 1 h, and supernatants were collected and stored at -80°C for later analysis. Protein concentration was determined by the BCA assay (Thermo Fisher Scientific). Protein was electrophoresed in 8-16% Tris-HCl polyacrylamide gel and transferred to a polyvinylidene difluoride membrane (Bio-Rad) for 90 min at 4 °C. After 1 h in TBS, 0.1% Tween-20 with 5% w/v nonfat dry milk, membranes were incubated overnight with primary antibodies against CD63 (1:1000; Biolegend), HSP70 (1:1000; Cell Signaling Technology), TSG101 (1:200; Servicebio), CD9 (1:500; Bioss), Calnexin (1:1000; Santa Cruz). After rinsing, blots were incubated with peroxidase-linked secondary antibodies, treated with ECL substrate, and signals were visualized using BioRad.

### iTRAQ proteomic analysis

HAs were grown to 80% confluency in Falcon T-125 flasks. Cells were treated by ultrasound stimulation as described. Exosomes were isolated from the supernatants by ultracentrifugation. Samples were stored at -80 °C until required. Proteins were extracted, the concentration was measured by the BCA assay, and the samples were analyzed by SDS-PAGE. Samples were reductively alkylated, and trypsin was used for enzymatic hydrolysis. The peptides were labeled with iTRAQ 8PLEX (SCIEX) and the equally mixed labeled peptides were pre-separated by UHPLC (Thermo SCIENTIFIC Vanquish) with C18 reversed-phase column (ACQUITY UPLC BEH C18 Column 1.7 µm, 2.1 mm×150 mm, Waters, USA). Subsequently, liquid chromatography-tandem mass spectrometry (LC-MS-MS) was performed for the identification and quantification of proteins. After searching for the Sequence or Mascot module in Proteome Discoverer Software 2.2, statistical and bioinformatic analyses of the results were performed.

### Amyloid-β oligomer

Aβ_42_ was dissolved in hexafluoro-2-isopropanol (HFIP) at 1 mg/mL^-1^ and stored at -20 °C. The amyloid-β peptide stock solutions were prepared in DMSO by vigorous vortexing and sonication 10 times for 3 s on ice, and finally filtering the solution through a 0.22 μm filter unit. SH-SY5Y cells seeded in a 96-well plate, Aβ_42_ peptides were added to the cells at a final concentration of 10 μM. 48 h after the addition of Aβ_42_, the medium was changed to fresh DMEM. US-HA-EXO at different protein concentrations were added to the Aβ_42_ intoxicated cells. Seventy-two hours later, the medium was replaced with fresh DMEM containing 10% CCK-8 assay solution, and CCK-8 assay was performed according to the manufacturer's protocol. The measurement of absorbance was performed at 450 nm with a BioTek Synergy plate reader.

### Uptake of oligomeric Aβ_42_ in the presence of exosomes by FACS

For the quantitative evaluation of exosomes on cellular uptake efficiency of FITC-Aβ_42_, SH-SY5Y cells were seeded in 24-well plate at 5×10^4^ per well and cultured overnight. Then cells were co-incubated with US-HA-Exo, and 2 μg/mL^-1^ FITC-Aβ_42_ for 0.5 h and 1 h at 37 °C. Then cells were washed by PBS and harvested and subjected to the analysis under a FACS (Beckmann, USA).

### Cellular colocalization of exosomes with Aβ_42_

PKH26, a red‐fluorescent lipophilic dye, was used for exosome labeling for *in vitro* trace. US-HA-exosomes were stained with PKH26 for 10 min, followed by washes and resuspension at a final concentration of 2 µg/mL. SH-SY5Y cells were seeded and cultured overnight in the Lab-Tek chamber slide. For CLSM observation, cells were incubated with oligomeric FITC-Aβ_42_ (10 μM) and PKH26-labeled exosomes for 4 h at 37 °C. At the end of the incubation, the medium was removed by washing with PBS three times. Cells were fixed with 4% paraformaldehyde solution and stained by 4′,6-diamidino-2-phenylindole (DAPI) for viewing and imaging. The chamber slides and exosomes were visualized with a Confocal Laser Microscope (Leica TCS SP5, Germany).

### Brain-targeted delivery of exosomes in mice assisted by FUS-BBB opening

To study FUS-BBB opening, the FUS transducer (1.0 MHz and 38 mm diameter) was driven by a function generator connected to a power amplifier. A removable cone filled with degassed water was employed to hold the transducer and guide the FUS beam into the brain. The BBB was opened at acoustic pressure of 0.6 MPa, with 10Hz pulse repetition frequency, at 10% duty cycle, and a total sonication duration of 60 s. The PLGA-lipid hybrid microbubbles were prepared as previously reported 24, with mean diameter of about 300 nm and concentration of about 1×10^9^ bubbles per mL. Microbubbles were intravenously injected just before ultrasound treatment. To confirm the successful BBB opening, the mice were administrated evans blue (EB) dye (30 mg/kg) via tail vein and sacrificed at 2 h after EB injection.

### Fluorescent labeling of exosomes by DiR

Near-IR (NIR) fluorescent lipophilic tracer DiR (1,1-dioctadecyl-3,3,3,3-tetramethylindotricarbocyanine iodide) was used to label the lipid bilayer of exosomes for in vivo tracking. For labeling, the exosome solution was incubated with 1 µM DiR at room temperature for 15 min. The unincorporated dyes were removed by using 100 KDa ultrafiltration tubes (Amicon Ultracel, Millipore) and washing with PBS. The exosome protein concentration was measured by using the BCA Protein Assay Kit (Pierce, ThermoFisher).

### *In vivo* imaging of FUS-assisted brain-targeted delivery of exosomes

For evaluating the blood-brain-barrier opening effect induced by ultrasound, the mouse head was depilated for detection by DiR. Each mouse was i.v. injected with 2 mg lyophilized microbubbles. After the microbubble-assisted BBB opening, BALB/c mice were anesthetized with isoflurane and injected intravenously with 60 µg DiR-labeled exosomes in 200 µL PBS. Brain-targeted delivery of DiR-exosomes in live mice was determined using IVIS SPECTRUM (Caliper Life Sciences). IVIS pictures were taken after 2 h and 4 h post-I.V. injection, with excitation/emission at 745/800 nm. Fluorescent background subtraction was performed and mice were imaged with identical instrument settings. Live Image 4.2 Software was used for quantitative analysis.

### Immunohistochemistry

Animals were deeply anesthetized, killed, and perfused intracardially with normal saline and fixed with 4% paraformaldehyde (PFA). Brains were placed in 15% sucrose in PBS for 6-12 h, then 30% sucrose in PBS until equilibrated. Brain coronal sections (10 μm) were cut with a cryostat (Leica CM1900), sections were used for immunohistochemistry. Slices were washed with PBS and fixed with 4% paraformaldehyde, permeabilized with 0.05% Triton X-100, then pre-incubated in 1% BSA solution for blocking. Slices were incubated overnight at 4 °C with the primary antibody solution (rabbit anti-amyloid-β antibody, Abcam; 1:500), mouse anti-NeuN (1:100, Sigma), rabbit anti-glial fibrillary acidic protein (GFAP) (1:250, Sigma) on a shaker. After washing with PBS, the sections were incubated with the secondary antibody (goat anti-rabbit Alexa Fluor 488 antibody, Abcam; 1:1000; goat anti-mouse Alexa Fluor 488 antibody, Abcam; 1:1000) for 2 h at room temperature. Sections were mounted in the vectashield mounting medium with DAPI (Vector laboratories). For the detection of amyloid plaques, brain tissue sections were stained in 0.1% thioflavin-S (Sigma) and rinsed with 70% ethanol. The brain tissue sections were also stained with hematoxylin and eosin (H&E) to assess the histological damage.

### Electron microscopy

HA cells were treated with ultrasound, 24 h later, cells were collected and fixed with 3% glutaraldehyde, after pre-embedding with agarose and postfixed in OsO_4_ in 0.1 M PBS (pH 7.4). After dehydration with ethanol, resin penetration, then cells were embedded in EMBed 812. The resin blocks were cut to 60-80nm thin on the ultra-microtome (Leica UC7), and the tissues were fished out onto the 150 meshes cuprum grids with formvar film, then stained with 2% uranium acetate saturated alcohol solution. The cuprum grids are observed under TEM (Hitachi, HT7800/HT7700) and take images.

### Statistical analysis

Statistical analysis was performed using Graphpad Prism Software. Data are presented as the means ± standard deviations (SD). Statistical analysis were conducted by unpaired two-tailed Student's t-test. Differences were considered significantly at ^*^P < 0.05; ^**^P < 0.01.

## Results

### Isolation and characterization of exosomes derived from insonated astrocytes

**Figure 1:**
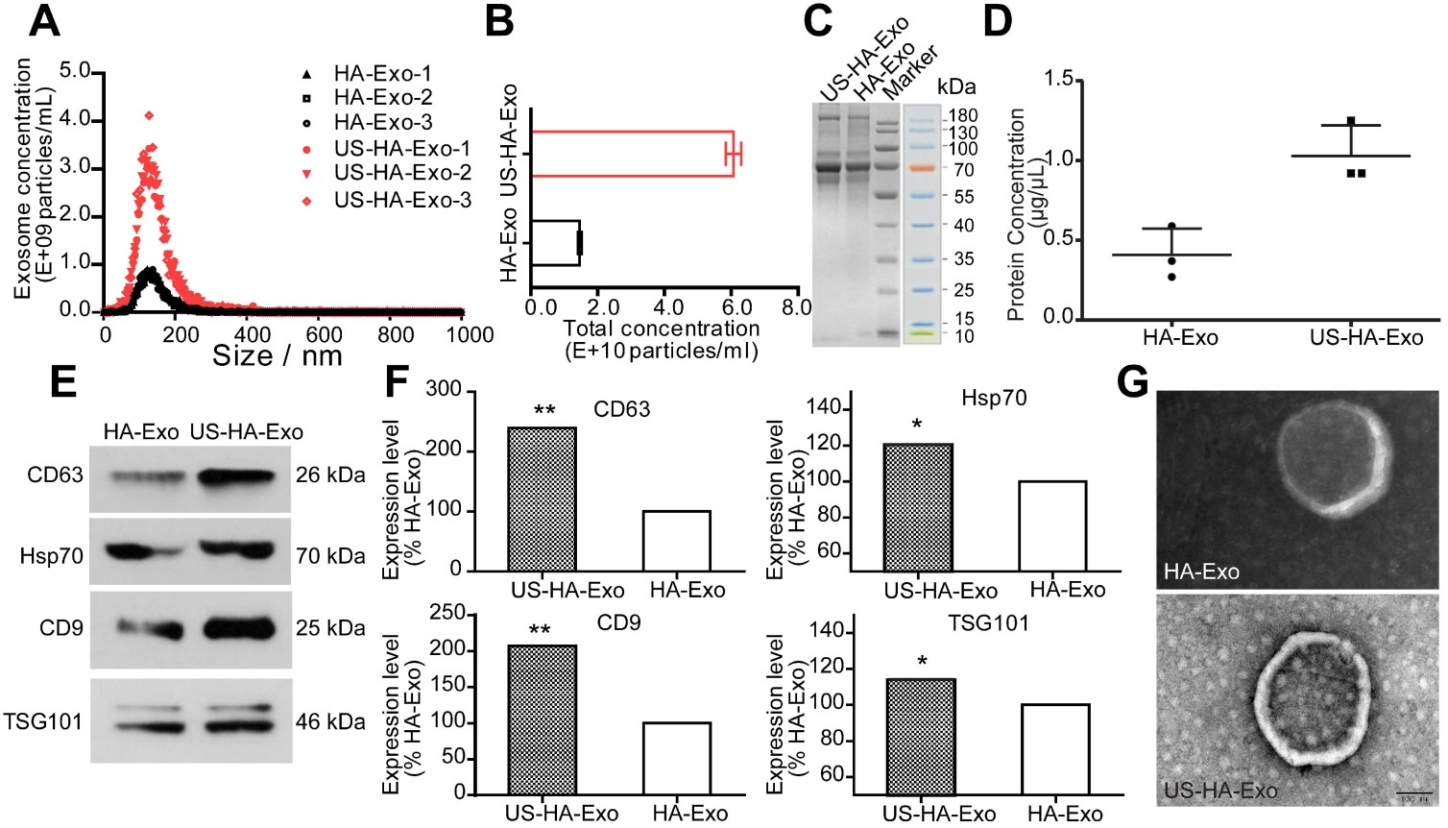
** Characterization and quantification of astrocyte-derived exosomes. (A)** NTA of ultrasound-stimulated astrocytes and astrocyte-derived exosomes showing the number and size distribution and** (B)** Total concentrations; total protein level of exosomes determined by SDS-PAGE **(C)** and BCA **(D)**. **(E, F)** Western blotting of biomarker proteins of exosomes from both ultrasound-stimulated astrocytes and astrocytes without any treatment. **(G)** Representative TEM of exosomes. Scale bars: 100 nm. * denotes significant difference compared with controls. Data are presented as means ± SD (n = 3). *P < 0.05; **P < 0.01.

To study the effects of ultrasound stimulation on exosome production, HA cells were cultured for *in vitro* experiments and treated with ultrasound as described in Methods. The size and number of the isolated US-HA-Exo (Figure 1A) were measured on a Marvern Nanosight, showing a relatively uniform distribution of particles with a size distribution of 131.4 ± 1.2 nm in diameter for HA-Exo and 131.7 ± 2.2 nm for US-HA-Exo. Total number of exosomes (Figure 1B) were 1.46 ± 0.06 E+10 particles/mL and 6.06 ± 0.23 E+10 particles/mL for HA-Exo and US-HA-Exo, respectively. Thus, ultrasound treatment resulted in a 4.14-fold increased production of exosomes. The CCK-8 assay was used to test HA cell proliferation after 72 h of ultrasound treatment. As shown in Figure S2, OD_450_ nm did not show a significant difference between control cells and ultrasound-treated cells. Thus, ultrasound treatment did no induce increased proliferation of astrocytes.

Total protein concentration was measured by SDS-PAGE (Figure 1C) and BCA analysis. Results for BCA assay (Figure 1D) was 0.41 ± 0.16 μg/μL (HA-Exo) and 1.03 ± 0.19 μg/μL (US-HA-Exo), demonstrating a significantly increased production of astrocyte-derived exosomes post-insonation. Western blotting confirmed that ultrasound stimulation of HA cells (US-HA-Exo) led to a significant increase in exosome marker proteins, such as CD63, HSP70, CD9, and Tsg101, compared with the control HA cells (Figure 1E-F). We also carried out Western blotting for calnexin, and cell lysates were used as positive control. It is clear from Figure S1, exosomes did not have calnexin, showing that there was no cytoplasmic contamination in the preparation. Furthermore, consistent with the NTA results (Figure 1A), electron microscopic analysis confirmed that exosomes secreted by US-HA cells were identical in size distribution and morphology (Figure 1G) to those produced by untreated HA cells.

### Cellular uptake of exosomes in SH-SY5Y cells

**Figure 2:**
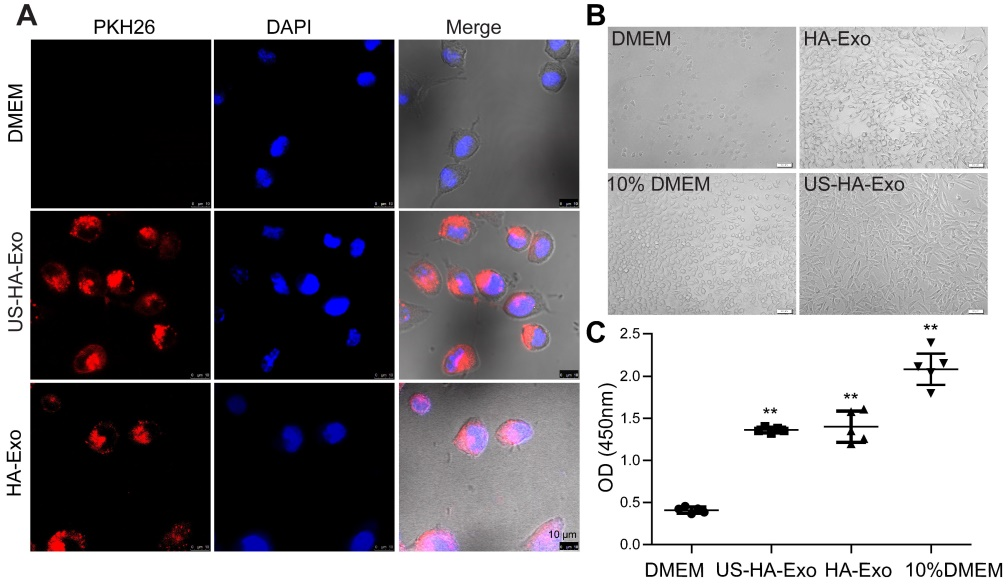
**Uptake of PKH26-labeled exosomes by SH-SY5Y cells. (A)** Confocal images of cultures exposed to HA-Exo, and US-HA-Exo for up to 4 h. Fluorescent signals are merged with transmission images (red = PKH26, blue = DAPI). Scale bar is 10 µm. **(B)** Images of SH-SY5Y cells were taken. **(C)** CCK-8 assays were performed.

Exosomes are known to be taken up by other cells by endocytosis, triggering cellular responses 25. We performed uptake studies with PKH26-labeled exosomes in human neuroblastoma SH-SY5Y cells, which were grown using a Lab-Tek chamber. Uptake of PKH26-labeled exosomes was evaluated with CLSM after 4 h incubation. As shown in Figure 2A, exosomes (red fluorescence) were internalized in SH-SY5Y cells rapidly and accumulated in the cytoplasm. After 48 h of incubation with exosomes, cell proliferation of serum-starved SH-SY5Y cells was tested by the CCK-8 assay. And morphological changes of SH-SY5Y cells were taken (Figure 2B). The results (Figure 2C), showing the values of 0.41 ± 0.036, 1.40 ± 0.18, 1.36 ± 0.033, and 2.08 ± 0.18 for DMEM, HA-Exo, US-HA-Exo, and 10% DMEM, respectively. Morphological images and CCK-8 assays demonstrated that both HA-Exo and US-HA-Exo increased proliferation of serum-starved SH-SY5Y cells.

### US-HA-Exo mitigate the toxicity of Aβ_42_ oligomers to SH-SY5Y cells

**Figure 3:**
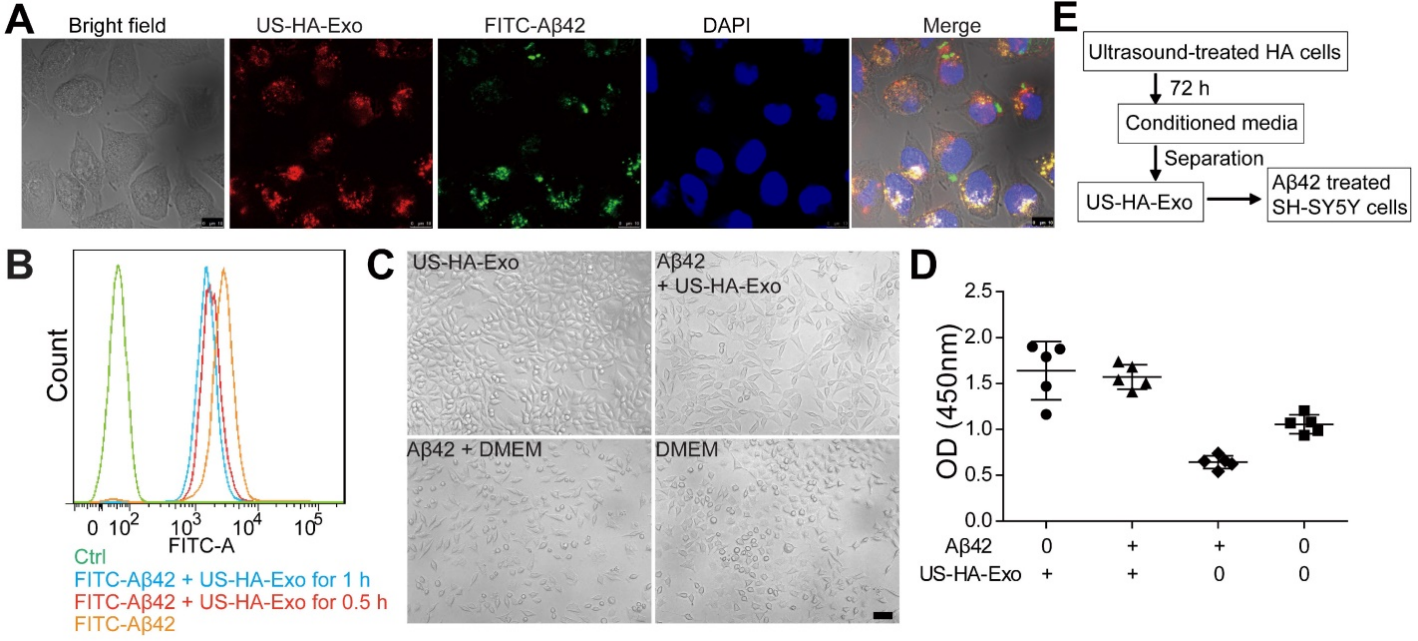
** Colocalization of exosomes with FITC-Aβ_42_ in SH-SY5Y cells, exerting neuroprotective effects against Aβ_42_ oligomers. (A)** Exosomes were labeled with PKH26 (red fluorescence), and observed by confocal laser microscopy, scale bar: 10 µm. **(B)** FACS-based internalization assay. **(C, D)** Effects of HA-Exo and US-HA-Exo on cell viability in human SH-SY5Y neuroblastoma cells treated with oligomeric Aβ_42_ or vehicle (DMEM). The cells were preincubated with 10 µM Aβ_42_ for 24 h prior to the addition of exosomes. The morphological pictures were taken, scale bar: 50 µm. The viability of the cultures was assessed by the CCK-8 assay 72 h after the addition of exosomes. **(E)** Schematic of exosome production, isolation and its cellular effects.

To test whether HA-exosomes could rescue the neurotoxic effects of Aβ_42_, cellular uptake and colocalization of exosomes were analyzed by CLSM and flow cytometry. Exosomes were visualized by staining with PKH26. SH-SY5Y cells were treated with 10 μM FITC-Aβ_42_ (green) and 50 μg US-HA-Exo (red). After incubation, the confocal micrograph of bright field and fluorescence images were acquired (Figure 3A). The yellow color indicated that exosomes could colocalize with oligomeric amyloid-β in the cytoplasm of neurons, demonstrating intracellular internalization of FITC-Aβ_42_.

Exosomes from astrocytes and 10 μM FITC-Aβ_42_ were added to neuroblastoma SH-SY5Y cells and incubated for 0.5 h and 1 h at 37 ºC. Subsequently, cells were washed with PBS twice, trypsinized, and detected by flow cytometry. FACS-based internalization assay showed that exosomes from astrocytes decreased the uptake of FITC-Aβ_42_ by SH-SY5Y cells (Figure 3B).

To investigate the protective potential of US-HA-exosomes against Aβ_42_ oligomers-induced neuronal toxicity, SH-SY5Y cells were exposed to 10 μM Aβ_42_ oligomers or vehicle for 24 h. Subsequently, the culture medium was replaced with fresh DMEM and exosomes were added to Aβ_42_ oligomers-treated SH-SY5Y cells for another 24 h. Representative bright-field images of the DMEM-, Aβ_42_-, and exosomes-exposed neurons are shown in Fig. 3C. The CCK-8 assay showed that Aβ_42_ oligomers decreased SH-SY5Y cell viability by 39.3% relative to DMEM controls (Figure 3D). The CCK8 assay results at OD450 nm were as follows: DMEM: 1.05 ± 0.1, Aβ_42_: 0.64 ± 0.07, US-HA-Exo: 1.64 ± 0.31 and Aβ_42_+US-HA-Exo: 1.57 ± 0.13. These results indicated that the exosomes rescued the cells from Aβ oligomers-induced toxicity. And Aβ ELISA for cell lysates was performed. Compared with Aβ_42_ exposed cells, a 26.3% decrease of Aβ concentration was found post Aβ_42_+US-HA-Exo treatment (Figure S3).

### FUS-assisted brain targeted delivery of exosomes

The BBB impedes the delivery efficiency of therapeutics. To enhance exosomes' brain-targeting abilities, BBB was non-invasively opened by applying low-intensity focused ultrasound (FUS) with microbubbles as previously reported 26. Mice were intravenously injected with exosomes immediately followed by FUS-mediated BBB opening.

**Figure 4:**
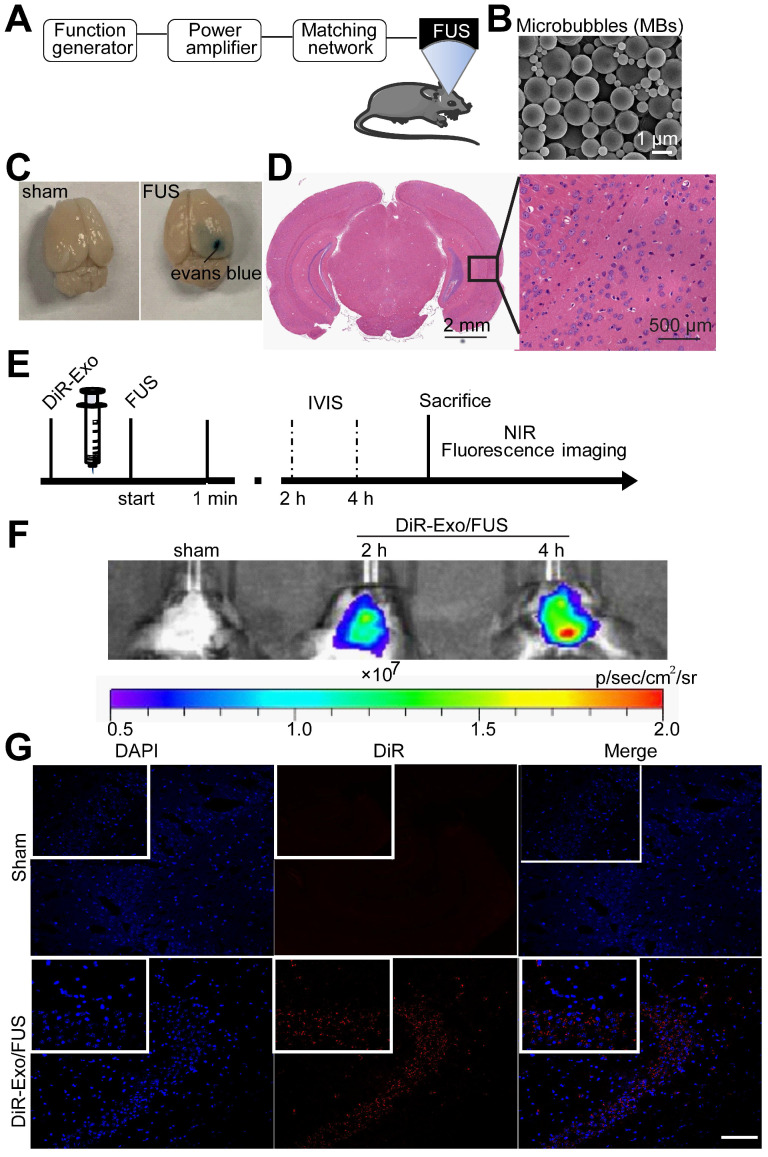
** Enhanced delivery efficiency of DiR-exosomes by FUS-BBB opening. (A)** Setup of FUS-BBB opening equipment. **(B)** SEM of microbubbles, scale bar: 1 µm. **(C)** BBB opening by FUS was indicated by evans blue dye.** (D)** H&E staining of brain post FUS treatment. **(E)*** In vivo* imaging scheme of FUS-BBB opening-assisted brain-targeted delivery of DiR-labeled exosomes. **(F)** IVIS imaging of brain accumulation of DiR-labeled exosomes after i.v. injection post FUS-BBB opening. **(G)** Brain accumulation of DiR-labeled exosomes after FUS-BBB opening demonstrated by CLSM pictures of brain slices, scale bar: 100 µm.

Normally Evans blue (EB) cannot pass through the BBB and, therefore, extravasation of EB in the brain tissue indicates alterations in BBB permeability. In this study, successful BBB opening was validated by extravasation of the EB dye (Figure 4C, right), while the BBB remained intact when mice were injected with saline instead of microbubbles (Figure 4C, left). EB staining indicated successful and localized BBB opening (Figure 4C). H&E staining of the brain showed no tissue damage or hemorrhage induced by FUS-BBB opening (Figure 4D). Thus, FUS-mediated BBB opening was demonstrated to be safe and effective.

To characterize the brain distribution of exosomes facilitated by the FUS-BBB opening, *in vivo* imaging of the brain was performed by an IVIS imaging system. BALB/c mice were anesthetized with isoflurane and injected intravenously with DiR-labeled exosomes immediately after the FUS-BBB opening. NIRF imaging was carried out at 2 h and 4 h post exosome injection. Figure 4F demonstrated a rapid uptake of exosomes in the brain when combined with FUS-BBB opening, consistent with our previous results of the enhanced brain accumulation of therapeutics 27. Two hours after intravenous injection, the majority of exosomes accumulated in the brain. We also detected the presence of exosomes in the mice brain tissue sections by fluorescence imaging of brain tissues. Fluorescence images of brain slices from the Exo/FUS group revealed a wide distribution of DiR-labeled exosomes (red fluorescence) in the mice brain (Figure 4G). These observations indicated that FUS-BBB opening enhanced exosome accumulation in the brain compared to the sham group. After GFAP staining for astrocytes, or NeuN staining for neurons, confocal laser microscopy was used to detect the localization of DiR-labeled exosomes in the brain. The results displayed in Figure S4 showing that exosomes were localized in the brain parenchyma, and mainly internalized in neurons, not in astrocytes.

### FUS-BBB opening-assisted exosome delivery augments Aβ clearance in APP/PS1 mice

**Figure 5:**
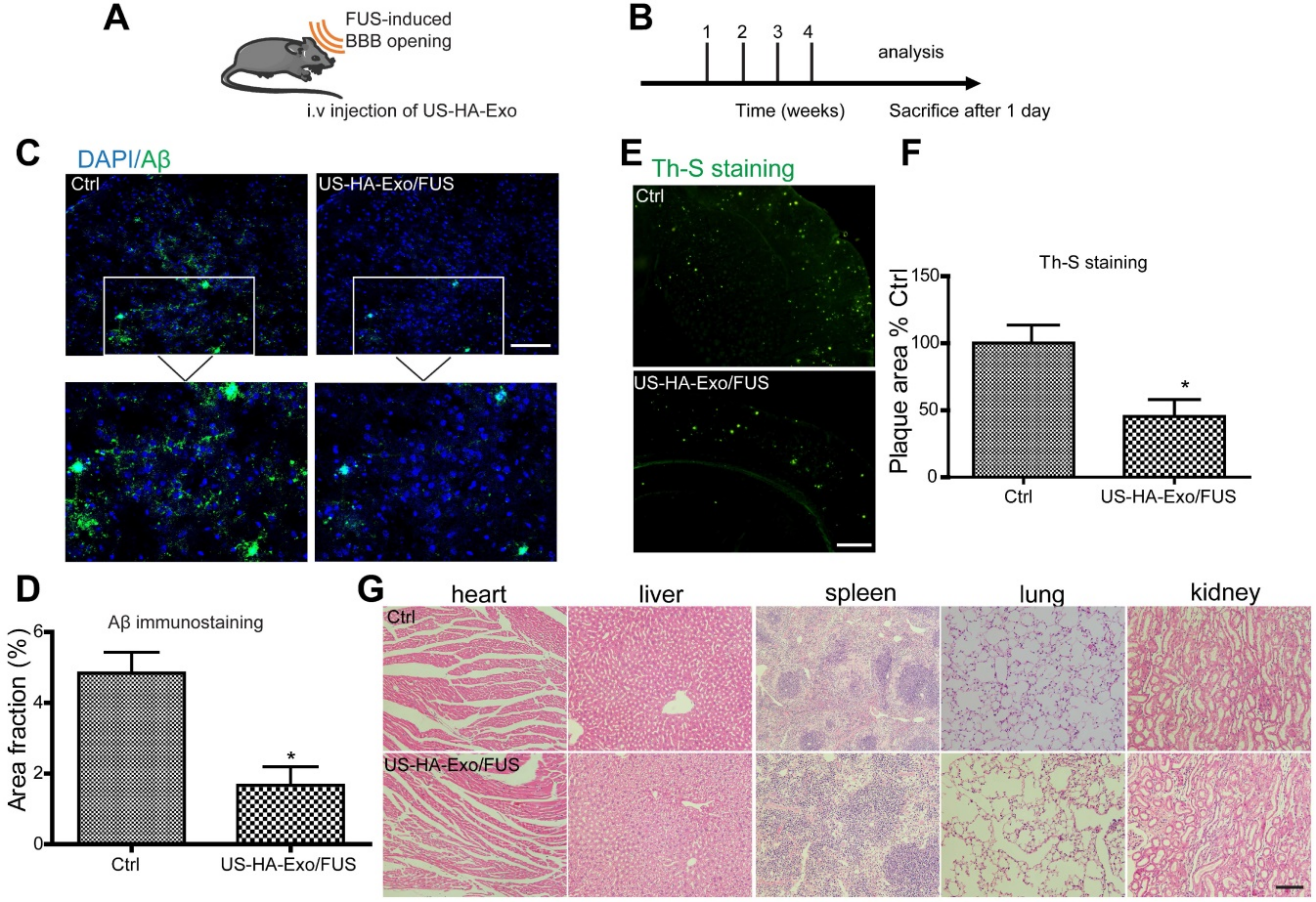
** Brain-targeted delivery of US-HA-Exo combined with FUS-mediated BBB opening. (A, B)** Schematic of FUS-BBB opening-assisted delivery of exosomes. **(C)** Brain sections obtained from 10-month-old APP/PS1 mice were immuno-stained for Aβ, scale bar: 100 µm. **(D)** The percentage area of positive amyloid-β stainning was quantified. **(E, F)** Aβ plaques in the brain were detected by thioflavin-S staining (scale bar: 100 µm), and quantified. **(G)** H&E staining of major organs of mice, scale bar: 100 µm.

Biodistribution of exosomes in mice after systemic delivery is affected by routes of injection or other factors 28; the BBB impedes the delivery efficiency of exosomes to the brain. To determine whether US-HA-Exo combined with FUS-BBB opening affected amyloid-β *in vivo*, immunofluorescent staining of amyloid-β and thioflavin-S staining of plaques were performed on brain slices after four weeks of treatment (Figure 5A, 5B). APP/PS1 mice treated with US-HA-Exo and FUS showed significantly reduced Aβ immunostaining (Figure 5C). Importantly, thioflavin-S staining revealed that the amyloid plaque deposition in treated APP/PS1 mice (US-HA-Exo/FUS) was also significantly lower than the control APP/PS1 mice (Figure 5E). Quantitative image analysis confirmed this statistically significant reduction for both Aβ immunostaining and thioflavin-S staining in US-HA-Exo/FUS-treated mice compared to APP/PS1 mice treated with saline (5D, 5F). H&E staining performed on major organs showed no damage after four weeks of treatment (Figure 5G).

### Analysis of iTRAQ-based quantitative proteomics of HA- and US-HA-exosomes

**Figure 6:**
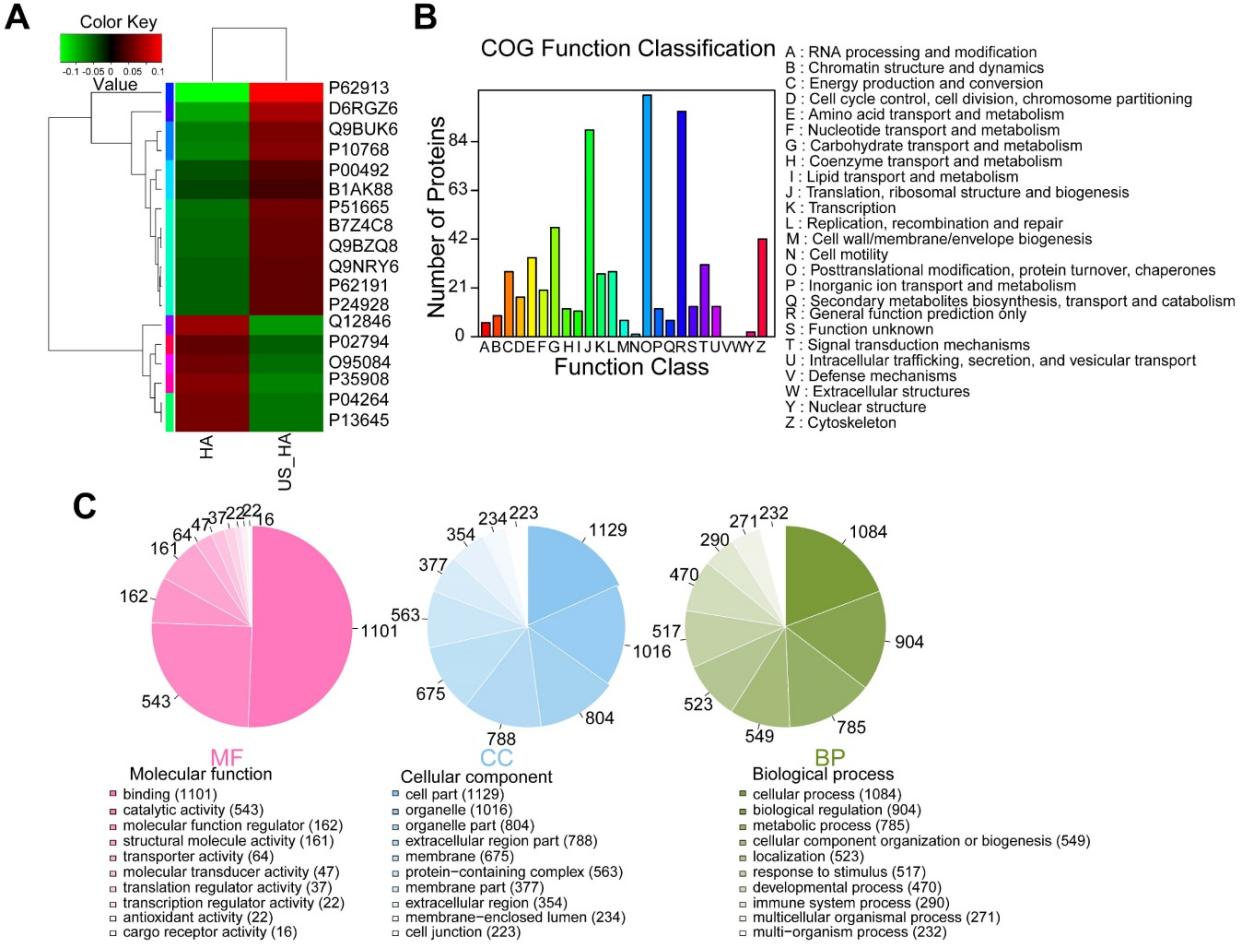
**Comparative proteomic analysis of HA-Exo with US-HA-Exo. (A)** Heatmap of HA-Exo- vs US-HA-Exo-regulated proteins. Each column represents a sample, and each row represents a protein. The colors in the diagram represent the relative expression of the protein in the group. Red represents a higher protein expression level in the sample, and the green represents a lower expression level. **(B)** Cluster of Orthologous Groups (COG) function classification. **(C)** Each pie chart has a different color representing a different GO Term, and its area represents the relative proportion of protein in the GO Term. The chart contains three pie charts; three rows from left to right represent three branches of GO, MF (molecular function, pink), CC (cell component, blue), and BP (biological process, green); each row is illustrated below with the corresponding GO classification term.

To investigate whether ultrasound treatment of astrocytes is reflected in exosome protein content, iTRAQ-based quantitative proteomic analysis was performed. HA-Exo and US-HA-Exo were isolated by ultracentrifugation and a total of 1226 proteins were quantified (Table S1). Among these proteins, 12 proteins were significantly upregulated and six proteins were significantly downregulated in US-HA-Exo compared to the control group (as determined by p<0.05 & Fold-Change (FC)<0.83 or FC>1.20. Table S2). The expression levels of 18 proteins identified in the US-HA-Exo group relative to the control group (HA-Exo) are displayed in a heatmap (Figure 6A). The iTRAQ-based proteomic results demonstrated that ultrasound-treated astrocytes released exosomes that exhibited specific proteomic alterations compared with control cells.

### Increases in number of multivesicular bodies (MVBs) in ultrasound-treated HA cells

**Figure 7:**
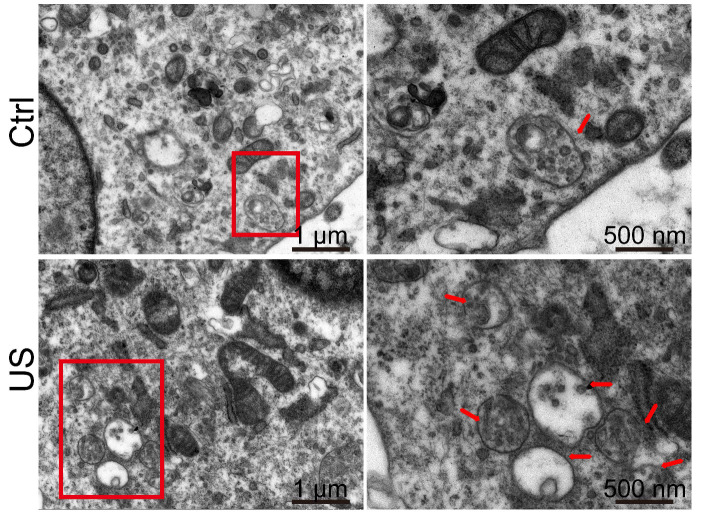
** Electron microscopy analysis of representative ultrasound-treated astrocytes (US) and untreated astrocytes (control).** MVBs, (red arrowhead) containing exosomes inside are evident in ultrasound-treated cells. Scale bars: 1 µm (left images), and 500 nm (right images).

Exosomes are formed as intraluminal vesicles (ILVs) by budding into early endosomes and multivesicular bodies (MVBs) 29. To gain further insight into, we performed electron microscopic analysis to investigate the number and morphology of MVBs and better understand the underlying mechanisms for increased exosome secretion in ultrasound-treated astrocytes. A dramatic increase of the number of MVBs (indicated by red arrows in Figure 7) per cell was observed by TEM compared with control cells, whereas the MVB morphology was unchanged.

## Discussion

Therapeutic ultrasound over a wide range of frequencies and pressures is currently used for treating many diseases 30, 31, and is a promising therapeutic approach for neuromodulation in AD therapy. Although ultrasound neuromodulation therapies have clear beneficial effects on neurodegenerative diseases 32, detailed mechanistic studies are needed to address various concerns. As the most abundant cell type in the brain, astrocytes may be centrally involved in exerting the neuro-modulatory effects. Astrocytes secrete a wide array of neurotransmitters, neuromodulators, and hormones, as well as metabolic and trophic factors, contributing to the gliocrine system 33. Emerging evidences show that exosomes secreted from astrocytes contain neuroprotective cargoes that could support the survival of neighboring neurons 34, 35, possibly providing neuroprotection in neurodegenerative diseases. Our findings suggested that exosomes secreted from astrocytes are protective against AD pathology. More importantly, ultrasound treatment could enhance the release of exosomes from astrocytes.

The upregulation of exosome release represents a potential therapeutic goal for neurodegenerative disorders with endosomal pathology. For example, dysfunctional oligodendrocyte exosome release is linked to multiple system atrophy 36. Recently, it was reported that increased Aβ levels in the brain might be the consequence of impaired exosome-mediated Aβ clearance pathway 6. APOE4-driven decreases in exosome levels in the post-mortem brains of neuropathologically healthy humans as well as in APOE mice in an age-dependent manner have been described 8. As previously reported, ultrasound stimulation upregulated BDNF production in astrocytes 37, 38, exerting protective effects against aluminum-induced AD rat model 39. Deficient exosome release was also observed in the mouse model of Huntington's disease 34.

AD is believed to be associated with a severely damaged clearing function of toxic proteins, such as oligomeric amyloids. It has been reported that increased exosome release may be an endogenous mechanism mitigating endosomal abnormalities in Downs Syndrome 10. It was also reported that the modulation of release of exosomes might alter the risk of AD 40, 41. Consistent with previous studies, herein, we confirmed that significantly increased exosome release by ultrasound treatment was beneficial for AD therapy. This finding offers a novel insight into ultrasound neuromodulation, suggesting that US-stimulated increased release of astrocyte exosomes exerts a neuroprotective effect in AD transgenic mice.

Recent data suggested that the release of extracellular vesicles could be enhanced by ultrasound combined with microbubbles 42, or nanodroplets 43. However, the biological application of this approach remained unclear, and there are currently no published reports linking the enhanced release of exosomes with ultrasound-mediated neuro-modulatory effects. The present study used ultrasound for astrocyte stimulation, even without microbubbles, resulting in a significantly enhanced exosome release from astrocytes. To the best of our knowledge, our study is the first to evaluate ultrasound-stimulated astrocyte-derived exosomes (US-HA-Exo), mitigating the neurotoxicity of amyloid-β oligomers. Our results imply that US-HA-Exo have the potential to reverse amyloid-β-induced cytotoxicity and provide neuroprotective effects *in vitro* and, when combined with FUS-induced BBB opening, cross the BBB and enable the clearance of amyloid-β plaques *in vivo*.

Furthermore, we performed a detailed proteomic analysis of exosomes following ultrasound treatment. The mechanistic insight into the enhanced exosome release upon insonation is lacking. It may be because ultrasound stimulation triggers Ca^2+^ influx in astrocytes 44, or the cavitation effect causes the upregulated release by plasma membrane deformation 45. Besides, protein expression was altered in astrocyte exosomes upon ultrasound stimulation. Ultrasound stimulation also upregulated MVB in US-HA cells compared with the control group. Since exosomes are derived from MVB 46, thus, our findings furnish significant new mechanistic insights into the ultrasound-enhanced release of exosomes. Also, astrocytic exosomes carry heat shock proteins (hsp) which are reported to reduce cellular toxicity of misfolded proteins, thus preventing neurodegeneration *in vitro* 35. Consistent with this finding, hsp70 protein expression was significantly increased in our study due to ultrasound-stimulated amplified exosome production.

Some proteins are highly expressed in US-HA-Exo compared with HA-Exo, such as P62191, P51665; they are multiprotein complexes involved in the ATP-dependent degradation of ubiquitinated proteins, which play an important role in the maintenance of protein homeostasis by removing misfolded proteins 47. Therefore, it may explain the ability of US-HA-Exo to remove misfolded proteins such as oligomer amyloid-β in this study. Tamboli et al. showed that enhancement of exosome generation, especially secretion of insulin-degrading enzyme (IDE) via exosomes, improved Aβ clearance ability 48. Yuyama et al. injected exosomes intracerebrally, resulting in reductions in Aβ pathology in mice, and exosomal glycosphingolipids were critical for the Aβ clearing activity 49.

It has been reported that cells are able to sense external stimulation, such as mechanical or optical, and transduce these stimuli into biochemical signals 50, triggering cellular repair pathways 51. Ultrasound stimulation upregulated BDNF production in astrocytes by activating calcium signaling pathways 37, and increasing protein levels of neurotrophic factors 38. Also, ultrasound treatment significantly attenuated the LPS-induced increase in Aβ and amyloid precursor protein (APP) expression in the hippocampus of LPS-treated mice 52. Consistent with previous results 53, our findings indicated that exosomes from astrocytes could mitigate *in vitro* toxicity of Aβ oligomers. As previously demonstrated 27, 54, 55, FUS-mediated BBB opening is a temporary, reversible, and safe method for brain-targeted exosomes delivery. In the present study, we have demonstrated that exosomes, assisted by the FUS-induced noninvasive and transient opening of BBB, exhibit Aβ removing effect in APP/PS1 mice.

## Conclusion

In summary, ultrasound may treat neurodegenerative diseases via amplified production of astrocyte exosomes, thereby mitigating the Aβ toxicity to neurons. Astrocyte-derived exosomes can decrease *in vitro* toxicity of Aβ and clear the Aβ plaques in AD mice. Therefore, we propose that the augmented exosome release by ultrasound may provide a new therapeutic modality for the treatment of AD.

## Supplementary Material

Supplementary figures and tables.

## References

[1] Sperling RA, Aisen PS, Beckett LA, Bennett DA, Craft S, Fagan AM (2011). Toward defining the preclinical stages of Alzheimer's disease: Recommendations from the National Institute on Aging-Alzheimer's Association workgroups on diagnostic guidelines for Alzheimer's disease. Alzheimers Dement.

[2] Zott B, Simon MM, Hong W, Unger F, Chen-Engerer HJ, Frosch MP (2019). A vicious cycle of beta amyloid-dependent neuronal hyperactivation. Science.

[3] Tarasoff-Conway JM, Carare RO, Osorio RS, Glodzik L, Butler T, Fieremans E (2015). Clearance systems in the brain-implications for Alzheimer disease. Nat Rev Neurol.

[4] Peng KY, Perez-Gonzalez R, Alldred MJ, Goulbourne CN, Morales-Corraliza J, Saito M (2018). Apolipoprotein E4 genotype compromises brain exosome production. Brain.

[5] Cataldo AM, Peterhoff CM, Troncosco JC, Gomez-Isla T, Hyman BT, Nixon RA (2000). Endocytic pathway abnormalities precede amyloid beta deposition in sporadic Alzheimer's disease and Down syndrome - Differential effects of APOE genotype and presenilin mutations. Am J Pathol.

[6] Abdullah M, Takase H, Nunome M, Enomoto H, Ito J, Gong JS (2016). Amyloid-beta Reduces Exosome Release from Astrocytes by Enhancing JNK Phosphorylation. J Alzheimers Dis.

[7] Dinkins MB, Dasgupta S, Wang G, Zhu G, Bieberich E (2014). Exosome reduction *in vivo* is associated with lower amyloid plaque load in the 5XFAD mouse model of Alzheimer's disease. Neurobiol Aging.

[8] Peng KY, Perez-Gonzalez R, Alldred MJ, Goulbourne CN, Morales-Corraliza J, Saito M (2019). Apolipoprotein E4 genotype compromises brain exosome production. Brain.

[9] Nuriel T, Peng KY, Ashok A, Dillman AA, Figueroa HY, Apuzzo J (2017). The Endosomal-Lysosomal Pathway Is Dysregulated by APOE4 Expression *in vivo*. Front Neurosci.

[10] Gauthier SA, Perez-Gonzalez R, Sharma A, Huang FK, Alldred MJ, Pawlik M (2017). Enhanced exosome secretion in Down syndrome brain - a protective mechanism to alleviate neuronal endosomal abnormalities. Acta Neuropathol Commun.

[11] Blandford SN, Galloway DA, Moore CS (2018). The roles of extracellular vesicle microRNAs in the central nervous system. Glia.

[12] Rajendran L, Bali J, Barr MM, Court FA, Kramer-Albers EM, Picou F (2014). Emerging roles of extracellular vesicles in the nervous system. J Neurosci.

[13] Sharma P, Mesci P, Carromeu C, McClatchy DR, Schiapparelli L, Yates JR 3rd (2019). Exosomes regulate neurogenesis and circuit assembly. Proc Natl Acad Sci U S A.

[14] Budnik V, Ruiz-Canada C, Wendler F (2016). Extracellular vesicles round off communication in the nervous system. Nat Rev Neurosci.

[15] Cui GH, Wu J, Mou FF, Xie WH, Wang FB, Wang QL (2018). Exosomes derived from hypoxia-preconditioned mesenchymal stromal cells ameliorate cognitive decline by rescuing synaptic dysfunction and regulating inflammatory responses in APP/PS1 mice. FASEB J.

[16] Yang Q, Nanayakkara GK, Drummer C, Sun Y, Johnson C, Cueto R (2017). Low-Intensity Ultrasound-Induced Anti-inflammatory Effects Are Mediated by Several New Mechanisms Including Gene Induction, Immunosuppressor Cell Promotion, and Enhancement of Exosome Biogenesis and Docking. Front Physiol.

[17] Mohammadjavadi M, Ye PP, Xia A, Brown J, Popelka G, Pauly KB (2019). Elimination of peripheral auditory pathway activation does not affect motor responses from ultrasound neuromodulation. Brain Stimul.

[18] Scarcelli T, Jordao JF, O'Reilly MA, Ellens N, Hynynen K, Aubert I (2014). Stimulation of hippocampal neurogenesis by transcranial focused ultrasound and microbubbles in adult mice. Brain Stimul.

[19] Leinenga G, Gotz J (2015). Scanning ultrasound removes amyloid-beta and restores memory in an Alzheimer's disease mouse model. Sci Transl Med.

[20] Burgess A, Dubey S, Yeung S, Hough O, Eterman N, Aubert I (2014). Alzheimer disease in a mouse model: MR imaging-guided focused ultrasound targeted to the hippocampus opens the blood-brain barrier and improves pathologic abnormalities and behavior. Radiology.

[21] Sweeney MD, Kisler K, Montagne A, Toga AW, Zlokovic BV (2018). The role of brain vasculature in neurodegenerative disorders. Nat Neurosci.

[22] Lipsman N, Meng Y, Bethune AJ, Huang YX, Lam B, Masellis M (2018). Blood-brain barrier opening in Alzheimer's disease using MR-guided focused ultrasound. Nat Commun.

[23] Mead BP, Mastorakos P, Suk JS, Klibanov AL, Hanes J, Price RJ (2016). Targeted gene transfer to the brain via the delivery of brain-penetrating DNA nanoparticles with focused ultrasound. J Control Release.

[24] Chen Y, Liang YB, Jiang P, Li F, Yu B, Yan F (2019). Lipid/PLGA Hybrid Microbubbles as a Versatile Platform for Noninvasive Image-Guided Targeted Drug Delivery. Acs Appl Mater Inter.

[25] Gruenberg J, Stenmark H (2004). The biogenesis of multivesicular endosomes. Nat Rev Mol Cell Biol.

[26] Burgess A, Shah K, Hough O, Hynynen K (2015). Focused ultrasound-mediated drug delivery through the blood-brain barrier. Expert Rev Neurother.

[27] Wu M, Chen W, Chen Y, Zhang H, Liu C, Deng Z (2018). Focused Ultrasound-Augmented Delivery of Biodegradable Multifunctional Nanoplatforms for Imaging-Guided Brain Tumor Treatment. Adv Sci.

[28] Wiklander OP, Nordin JZ, O'Loughlin A, Gustafsson Y, Corso G, Mager I (2015). Extracellular vesicle *in vivo* biodistribution is determined by cell source, route of administration and targeting. J Extracell Vesicles.

[29] Kowal J, Tkach M, Thery C (2014). Biogenesis and secretion of exosomes. Curr Opin Cell Biol.

[30] Lin Z, Meng L, Zou J, Zhou W, Huang X, Xue S (2020). Non-invasive ultrasonic neuromodulation of neuronal excitability for treatment of epilepsy. Theranostics.

[31] Zhou W, Wang J, Wang K, Huang B, Niu L, Li F (2017). Ultrasound neuro-modulation chip: activation of sensory neurons in Caenorhabditis elegans by surface acoustic waves. Lab Chip.

[32] Ye J, Tang SY, Meng L, Li X, Wen XX, Chen SH (2018). Ultrasonic Control of Neural Activity through Activation of the Mechanosensitive Channel MscL. Nano Lett.

[33] Verkhratsky A, Matteoli M, Parpura V, Mothet JP, Zorec R (2016). Astrocytes as secretory cells of the central nervous system: idiosyncrasies of vesicular secretion. EMBO J.

[34] Hong Y, Zhao T, Li XJ, Li S (2017). Mutant Huntingtin Inhibits alphaB-Crystallin Expression and Impairs Exosome Secretion from Astrocytes. J Neurosci.

[35] Nafar F, Williams JB, Mearow KM (2016). Astrocytes release HspB1 in response to amyloid-beta exposure *in vitro*. J Alzheimers Dis.

[36] Yu Z, Shi M, Stewart T, Fernagut PO, Huang Y, Tian C (2020). Reduced oligodendrocyte exosome secretion in multiple system atrophy involves SNARE dysfunction. Brain.

[37] Liu SH, Lai YL, Chen BL, Yang FY (2017). Ultrasound Enhances the Expression of Brain-Derived Neurotrophic Factor in Astrocyte Through Activation of TrkB-Akt and Calcium-CaMK Signaling Pathways. Cereb Cortex.

[38] Yang FY, Lu WW, Lin WT, Chang CW, Huang SL (2015). Enhancement of Neurotrophic Factors in Astrocyte for Neuroprotective Effects in Brain Disorders Using Low-intensity Pulsed Ultrasound Stimulation. Brain Stimul.

[39] Lin WT, Chen RC, Lu WW, Liu SH, Yang FY (2015). Protective effects of low-intensity pulsed ultrasound on aluminum-induced cerebral damage in Alzheimer's disease rat model. Sci Rep.

[40] Yuyama K, Sun H, Mitsutake S, Igarashi Y (2012). Sphingolipid-modulated exosome secretion promotes clearance of amyloid-beta by microglia. J Biol Chem.

[41] Yuyama K, Sun H, Mikami D, Mioka T, Mukai K, Igarashi Y (2020). Lysosomal-associated transmembrane protein 4B regulates ceramide-induced exosome release. FASEB J.

[42] Yuana Y, Jiang L, Lammertink BHA, Vader P, Deckers R, Bos C (2017). Microbubbles-Assisted Ultrasound Triggers the Release of Extracellular Vesicles. Int J Mol Sci.

[43] Paproski RJ, Jovel J, Wong GK, Lewis JD, Zemp RJ (2017). Enhanced Detection of Cancer Biomarkers in Blood-Borne Extracellular Vesicles Using Nanodroplets and Focused Ultrasound. Cancer Res.

[44] Oh SJ, Lee JM, Kim HB, Lee J, Han S, Bae JY (2019). Ultrasonic Neuromodulation via Astrocytic TRPA1. Curr Biol.

[45] Fomenko A, Neudorfer C, Dallapiazza RF, Kalia SK, Lozano AM (2018). Low-intensity ultrasound neuromodulation: An overview of mechanisms and emerging human applications. Brain Stimul.

[46] Denzer K, Kleijmeer MJ, Heijnen HF, Stoorvogel W, Geuze HJ (2000). Exosome: from internal vesicle of the multivesicular body to intercellular signaling device. J Cell Sci.

[47] Kanayama HO, Tamura T, Ugai S, Kagawa S, Tanahashi N, Yoshimura T (1992). Demonstration that a human 26S proteolytic complex consists of a proteasome and multiple associated protein components and hydrolyzes ATP and ubiquitin-ligated proteins by closely linked mechanisms. Eur J Biochem.

[48] Tamboli IY, Barth E, Christian L, Siepmann M, Kumar S, Singh S (2010). Statins promote the degradation of extracellular amyloid {beta}-peptide by microglia via stimulation of exosome-associated insulin-degrading enzyme (IDE) secretion. J Biol Chem.

[49] Yuyama K, Sun H, Sakai S, Mitsutake S, Okada M, Tahara H (2014). Decreased amyloid-beta pathologies by intracerebral loading of glycosphingolipid-enriched exosomes in Alzheimer model mice. J Biol Chem.

[50] Wozniak MA, Chen CS (2009). Mechanotransduction in development: a growing role for contractility. Nat Rev Mol Cell Biol.

[51] Stewart MP, Sharei A, Ding X, Sahay G, Langer R, Jensen KF (2016). *In vitro* and *ex vivo* strategies for intracellular delivery. Nature.

[52] Chen TT, Lan TH, Yang FY (2019). Low-Intensity Pulsed Ultrasound Attenuates LPS-Induced Neuroinflammation and Memory Impairment by Modulation of TLR4/NF-kappaB Signaling and CREB/BDNF Expression. Cereb Cortex.

[53] Xin H, Wang F, Li Y, Lu QE, Cheung WL, Zhang Y (2017). Secondary Release of Exosomes From Astrocytes Contributes to the Increase in Neural Plasticity and Improvement of Functional Recovery After Stroke in Rats Treated With Exosomes Harvested From MicroRNA 133b-Overexpressing Multipotent Mesenchymal Stromal Cells. Cell Transplant.

[54] Pan M, Zhang Y, Deng Z, Yan F, Hong G (2018). Noninvasive and Local Delivery of Adenoviral-Mediated Herpes Simplex Virus Thymidine Kinase to Treat Glioma Through Focused Ultrasound-Induced Blood-Brain Barrier Opening in Rats. J Biomed Nanotechnol.

[55] Meng Y, Pople CB, Lea-Banks H, Abrahao A, Davidson B, Suppiah S (2019). Safety and efficacy of focused ultrasound induced blood-brain barrier opening, an integrative review of animal and human studies. J Control Release.

