# Supplementary material for: Ultrasound-mediated augmented exosome release from astrocytes alleviates amyloid-β-induced neurotoxicity
Source: Theranostics. 2021 Feb 25;11(9):4351–62. doi: 10.7150/thno.52436 (PMC7977450; doi:10.7150/thno.52436)
Supplement: Supplementary file 1 — Supplementary figures and tables. [file thnov11p4351s1.pdf]

Supporting imformation

Ultrasound-mediated augmented exosome release from astrocytes alleviates amyloid-β-induced neurotoxicity

Zhiting Deng[1], Jieqiong Wang[1], Yang Xiao[1], Fei Li[1], Lili Niu[1], Xin Liu[1], Long Meng*[1], Hairong Zheng*[1]

[1]Paul C.Lauterbur research center for biomedical imaging, Shenzhen Institutes of Advanced Technology, Chinese Academy of Sciences, Shenzhen, People's Republic of China

1068 Xueyuan Avenue, Shenzhen University Town, Shenzhen, 518055, China

E-mails: long.meng@siat.ac.cn or hr.zheng@siat.ac.cn

Materials and Methods

Protein extraction

Make fresh extraction buffer (20 mM Tris-HCl (pH 7.5), 150 mM NaCl, 1 mM Na_2EDTA, 1mM EGTA, 1% Triton, 2.5 mM sodium pyrophosphate, 1 mM beta-glycerophosphate, 1 mM Na_3VO_4, 1 µg/ml leupeptin, 1 tab of complete EDTA-free Mini protease inhibitor, 1 mM PMSF). Brain tissue was homogenized in 4×(w/v) extraction buffer on ice, and the solution was centrifuged at 12000×g for 1 h at 4 °C, supernatant was obtained and stored at -80℃ for futureuse. Total protein concentration was determined by BCA assay.

ELISA

Aβ levels were determined using a sandwich ELISA. The kits for $A\beta_{42}$ were obtained from Jianglai (Shanghai, China). After protein extraction, Aβ standards and samples were tested duplicate according to manufacturer's protocol.

Results

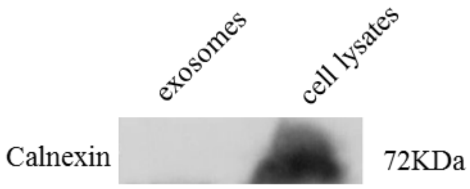

Figure S1. Western blotting of Calnexin, and cell lysates were used as positive control. Exosomes didn't have calnexin, showing that there was no cytoplasmic contamination in the preparation.

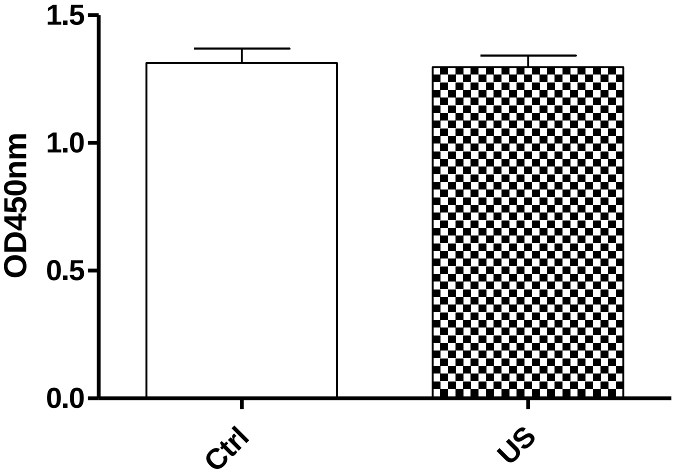

Figure S2. The CCK-8 assay was used to test HA cell proliferation after 72h of ultrasound treatment. As shown in Figure S2, OD450 nm did not show a significant difference between control cells and ultrasound-treated cells. Thus, ultrasound treatment did no induce increased proliferation of astrocytes.

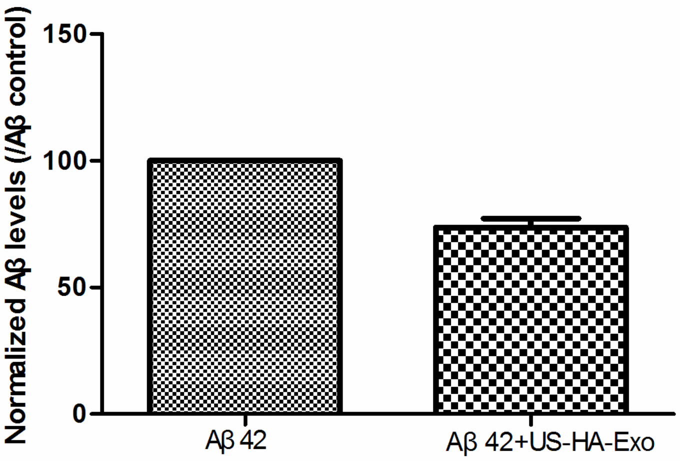

Figure S3. Aβ ELISA were performed of cell lysates to test intracellular Aβ.

Compared with control cells, a 26.3% decrease of Aβ concentration was found in the Aβ42+US-HA-Exo group as presented in Figure S3.

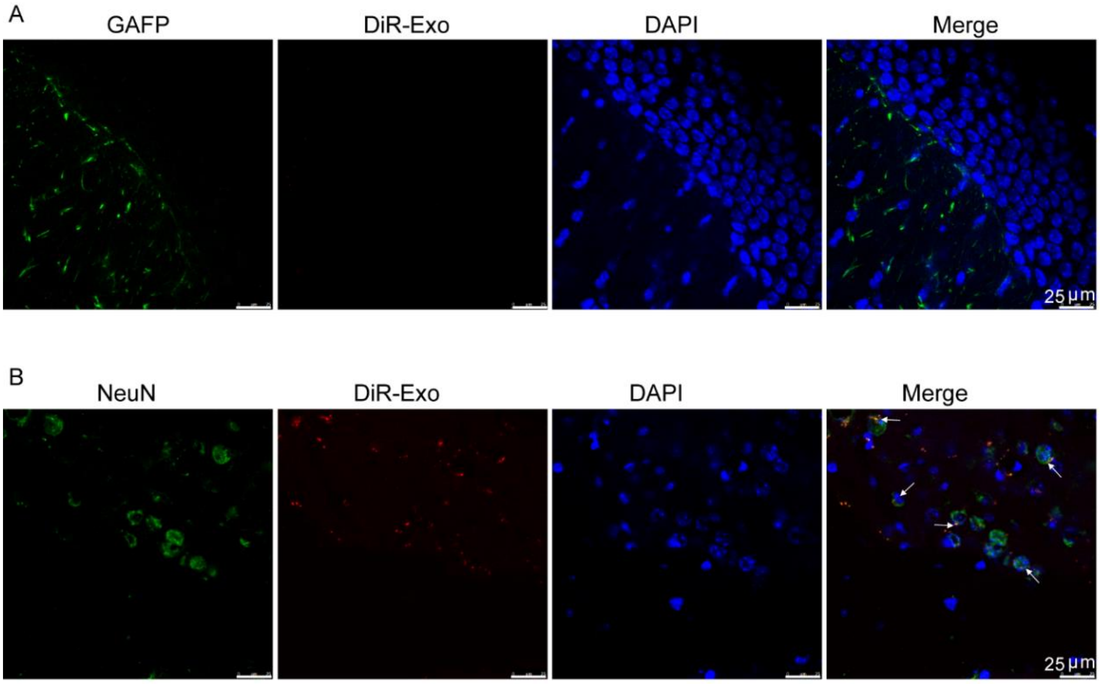

Figure S4. Double staining of GFAP and NeuN to detect the location of exosomes. After GFAP staining for astrocytes, or NeuN staining for neurons, confocal laser microscopy was used to detect the localization of DiR-labeled exosomes in the brain. The results displayed in Figure S4 show that exosomes were localized in the brain parenchyma, and mainly internalized in neurons, not in astrocytes.

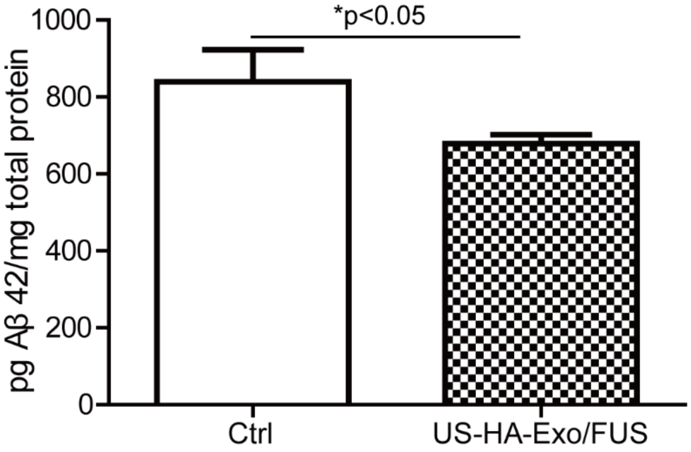

Figure S5. APP/PS1 mice were treated with US-HA-Exo and FUS-BBB for three weeks. Subsequently, brains were harvested, proteins were extracted, and Aβ ELISA was performed to measure the levels of Aβ. The results presented in Figure S5 revealed

a 19.15% decrease of Aβ42 in mouse brains in the US-HA-Exo/FUS-treated group compared to the control group (t-test, $P < 0.05$).

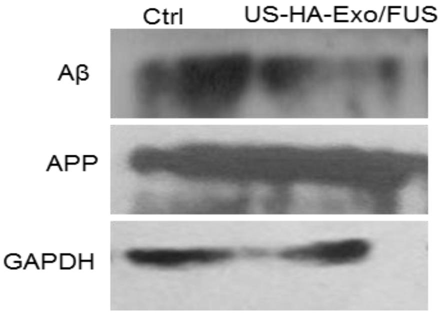

Figure S6. Western blotting for both APP and Aβ was carried out (Figure S6). Results showed that the expression level of APP did not change after treatment. However, decreased expression of Aβ proteins post US-HA-exo/FUS was evident by Western blotting.

Table S1. Statistical analysis of differential protein

The P-value of a significant difference between samples was calculated by t-test function in R language. The screening standard of significant difference in protein expression was $p < 0.05$ &Fold-Change (FC)$<$0.83 or FC$>$1.20.

Samples	Total number of proteins	Differential protein number	up regulated number of proteins	down regulated number of proteins
US-HA *vs* HA	1226	18	12	6

Table S2. List of up- and down-regulated protein expression between HA-Exo and US-HA-Exo

UniProt ID	Protein	Full name	FC(US-HA/HA)	Expression
P62191	PSMC1	26S proteasome regulatory subunit 4	1.287	up
P51665	PSMD7	26S proteasome non-ATPase regulatory subunit 7	1.329	up
Q9BUK6	MSTO1	Protein misato homolog 1	1.375	up
P00492	HPRT1	Hypoxanthine-guanine phosphoribosyltransferase	1.238	up
D6RGZ6	VCAN	Versican core protein	1.528	up
P10768	ESD	S-formylglutathione hydrolase	1.408	up
B7Z4C8	RPL31	60S ribosomal protein L31	1.303	up
P24928	POLR2A	DNA-directed RNA polymerase II subunit RPB1	1.272	up
Q9NRY6	PLSCR3	Phospholipid scramblase 3	1.280	up
Q9BZQ8	NIBAN1	Protein Niban 1	1.301	up
P13645	KRT10	Keratin, type I cytoskeletal 10	0.737	down
P04264	KRT1	Keratin, type II cytoskeletal 1	0.737	down
P35908	KRT2	Keratin, type II cytoskeletal 2 epidermal	0.711	down

P02794	FTH1	Ferritin heavy chain	0.786	down
O95084	PRSS23	Serine protease 23	0.761	down
Q12846	STX4	Syntaxin-4	0.673	down
